# The Differential Anti-HIV Effect of a New Humic Substance-Derived Preparation in Diverse Cells of the Immune System

**DOI:** 10.32607/20758251-2019-11-2-68-76

**Published:** 2019

**Authors:** G.V. Kornilaeva, A.E. Siniavin, A. Schultz, A. Germann, C. Moog, H. von Briesen, A.S. Turgiev, E.V. Karamov

**Affiliations:** Gamaleya Center for Epidemiology and Microbiology. Gamaleya Str.1 8, Moscow, 123098, Russia; Shemyakin-Ovchinnikov Institute of Bioorganic Chemistry, Miklukho-Maklaya Str. 16/10, Moscow GSP-7, 117997, Russia; Fraunhofer Institut fuer Biomedizinische Technik (IBMT), Joseph-von-Fraunhofer-Weg 1, 66280 Sulzbach, Germany; INSERM U1109, Fédération Hospitalo-Universitaire (FHU) OMICARE, Fédération de Médecine Translationnelle de Strasbourg (FMTS), Université de Strasbourg, 4 Rue Blaise Pascal, Strasbourg 67000 , France; Immunomica LLC, Novaya Basmannaya Str. 12, bldg. 2, ste. 103, Moscow, 107078, Russia

**Keywords:** humic derivative, suppression of HIV infection, CD4-positive T lymphocytes, macrophages, dendritic cells, T- and M-tropic HIV variants

## Abstract

The anti-HIV activity of a new humic substance-derived preparation has been studied in individual pools of immune cells (CD4+ T lymphocytes, macrophages, dendritic cells). Near-complete inhibition of the HIV infection (by more than 90%) was achieved by treating each of the abovementioned cell types with non-toxic concentrations of the preparation. The inhibitory effect demonstrates the possibility of preventing the depletion of a significant portion of functionally important immune cells. A comparative study of infection inhibition in individual cell pools has allowed us to reveal the differences in the preparation’s effectiveness in each of the cell populations. A R5-tropic HIV-1 infection in macrophages exhibited maximum sensitivity to the preparation: 90% and 50% inhibition of the infection were observed in the presence of concentrations as low as 1.4 and 0.35 μg/ml, respectively. A 15- and 19-fold higher concentration was required to achieve the same extent of inhibition in dendritic cells infected with the same strain. The effectiveness of the drug in CD4 + T lymphocytes is quite comparable to its effectiveness in macrophages. The drug is universally effective for both the T- and M-tropic variants of HIV-1.

## INTRODUCTION

Monocytes, macrophages, and dendritic cells, which constitute the cellular component of innate immunity and the first line of defense against pathogens [1-5], are the targets of HIV. The virus attacks the CD4-, CCR5-, and CXCR4-expressing cells present – depending on the invasion route – in the mucosa, blood, or lymphoid tissues. The transmission rate depends on both the expression level of the cellular surface receptors and the viral strain [6, 7]. A high viral production is commonly observed in CD4-positive T cells activated during acute infection [8]. Nevertheless, viral replication, in addition, takes place in quiescent CD4-positive memory cells, which also contain latently integrated or pre-integrated proviral DNA [9]. Cells harboring the viral genome represent a part of the infection reservoir that persists in the organism and may be retained for up to four years [9]. It is believed that quiescent CD4-positive memory cells are the first to be infected, and that the viruses involved are predominantly of the R5 phenotype [10-12]. This may be due to intergin LFA-1 expression [13]. The increased ability of R5 viruses to replicate may also explain their prevalence during an acute or primary HIV infection [14].

The pathogenetic process of HIV infection also involves antigen-presenting cells, macrophages and dendritic cells [15-18] which possess virus-specific receptors and are capable of actively circulating in the blood and penetrating various lymphoid and non-lymphoid tissues. Dendritic cells are prototypical antigen-presenting cells with an extraordinary capacity for recognizing and processing antigens [19]. When immature, dendritic cells show increased ability to phagocytose, while their functional pattern changes upon maturation and they exhibit preferential cytokine-producing activity. Expression of CCR5 decreases in the course of maturation, whereas that of CXCR4 increases, which may lead to the infection of dendritic cells by viruses of both phenotypes [18]. In addition, Langerhans cells (epidermal dendritic cells) are capable of taking up HIV (without subsequent viral production) via a C-type lectin: langerin. Maturation is associated with a decrease in viral production, which may be 10 to 100 times lower in mature as compared to immature dendritic cells [20, 17]. Most researchers agree that dendritic cells are not active viral producers; rather, their major role (favored by a pronounced capacity for migration) is to traffic HIV from the entry sites to CD4-positive T cells, thus promoting fast virus dissemination throughout the organism. Macrophages are also amenable to HIV infection [21, 22]. Macrophage-tropic R5 viruses replicate in macrophages derived from peripheral blood mononuclear cells, as well as in CD4-positive lymphocytes [23]. Macrophages also vary in their ability to produce the virus. Viral production in infected macrophages is frequently of low intensity, and the virus is found sequestered in intracellular vacuoles. The reduced viral replication may be due to the intracellular mechanisms of nonspecific defense. R5 viruses usually exhibit no cytopathic effect, but some isolates – particularly those detected at late stages of the disease – propagate in macrophages to high titers and may induce cytopathic effects [24, 25]. In the majority of cases, the death of the infected macrophages is a result of necrosis. Of particular importance to the pathogenesis of the HIV infection is the ability of macrophages to serve as a viral reservoir that is resistant to antiretroviral therapies and cannot be recognized by the cytotoxic lymphocytes of the immune system (due to the absence of active viral reproduction and synthesis of virus-specific proteins, respectively).

On the whole, the literature data indicate that the permissiveness of immune cells to HIV largely depends on their stage of maturation/differentiation and functional polarization. Both the level of cytokine production and the receptor repertoire are known to be affected by those processes. The infection-induced exhaustion of the pool of HIV-sensitive immune cells ultimately jeopardizes the buildup of the immune response.

Studies of the major target cells involved in HIV pathogenesis (such as peripheral blood mononuclear cells, macrophages, and dendritic cells) constitute an invariable component of the current requirements that need to be met in designing the assessment of new antivirals. This approach allows one to determine (a) the efficacy of the compounds under study in each relevant cell population and (b) the probability of affecting the course of the pathogenetic process (and ultimately optimize the therapeutic regimen) by administering those compounds.

In this work, we report on studies of a new anti-HIV preparation, a solubilized butanol fraction of humic substances (SBF-HS). Mass spectrometry data and the results of an elemental analysis indicate that the fraction contains 52.7% (w/w) and 37.1% (w/w) carbon and oxygen, respectively, but considerably lower amounts of nitrogen (4.3% w/w) and sulfur (2.1% w/w) [26, 27]. It should be noted that an elemental analysis of humic substances isolated from the same source using the same isolation and fractionation methods yields reproducible results. High-performance liquid chromatography (HPLC) data demonstrate that the preparation is characterized by a high content of hydrophobic aromatic fragments (74%), which was confirmed by 13C NMR. The hydrogen-to-carbon atomic ratio (H/C) is 0.8; the oxygen-to-carbon atomic ratio (O/C) is 0.53 [28-30].

It is known that humic substances form as a result of the decay of dead organisms and are among the vast reservoirs of organic carbon. SBF-HS is an ecologically sound and safe product exhibiting antitumor, antifungal, antibacterial, and antiviral activities. In addition, it stimulates hemopoiesis and acts as an immunomodulator, a powerful antioxidant, and an efficient hepatoprotector. SBF-HS stimulates cell-mediated immunity (particularly in inflammatory foci), accelerates the regeneration of wounds, burns, and ulcers of skin and mucosa, and lacks toxicity and allergenicity [31-33].

## MATERIALS AND METHODS

**SBF-HS**

Lignin-containing solid waste resulting from the processing of vegetable feedstock was used as a starting material for obtaining humic substances. The material was subjected to oxidative alkaline hydrolysis, followed by separation of the liquid phase, which was further acidified, and the resulting solid residue was isolated, washed, and dried. The intermediate product thus obtained was extracted with ethyl acetate. The resulting solid residue was extracted with *n*-butanol. In order to isolate and solubilize the biologically active fractions exerting antiviral effects, *n*-butanol was evaporated and the solid residue was purified by repeated precipitation from the alkaline solution (using concentrated hydrochloric acid). The fractions were standardized by the characteristic absorption bands in the infrared region (presence of specific absorption bands in the region 2–10 μm) and molar mass distribution (showing a maximum in the vicinity of 7,000 Da). The yield of the target product, the solubilized butanol fraction of humic substances (SBF-HS), approximated 40%.

**Physicochemical methods for analyzing SBF-HS**

*Elemental analysis*. Samples of the test compound were brought to complete dissolution by heating to 80–100°C in 2 ml of nitric acid, with a few drops of hydrogen peroxide added, for 3 hours. The solution was analyzed by atomic emission spectrometry with inductively coupled argon plasma (ICAP-9000; Thermo Jarrell Ash, USA).

*GC-MS analysis*. An accurately weighed sample of SBF-HS was adjusted to full air-dry weight and treated with 5 ml of toluene in an ultrasonic bath. The extracts were filtered, dried over anhydrous sodium sulfate, and evaporated in a nitrogen current. Some samples were methylated. An aliquot of the sample was analyzed by GC-MS on an equipment complex consisting of an HP5890A gas chromatograph, an HP5988A mass spectrometer, and an HP59970C data processing system (Hewlett-Packard, USA). The sample components were identified using the Wiley mass spectral library.

*NMR spectroscopy *was performed using a Bruker Avance 400 MHz NMR spectrometer.

**Cell lines**

CEM-SS, an immortalized line cloned from a human T4 lymphoblastoid cell line by adhesion using poly-L-lysine and characterized by increased ability of virus-induced syncytium formation and fusogenic activity, is widely used to study HIV and its inhibitors (NIH AIDS Reagent Program No. 776, USA). The cells were maintained in a RPMI-1640 medium (Sigma, USA) supplemented with 10% fetal calf serum (Sigma, USA) and 2 mM L-glutamine (Sigma, USA.).

Peripheral blood mononuclear cells (PBMCs) were isolated from the EDTA-anticoagulated blood of HIV-seronegative donors by Ficoll® Paque Plus (GE Healthcare Worldwide, USA) density gradient centrifugation. HIV was inoculated into mitogen-stimulated PBMC, which were obtained by culturing in the presence of 5 μg/ml phytohemagglutinin (PHA; Sigma, USA) for 3 to 4 days. The infected cells were maintained in a RPMI 1640 growth medium supplemented with 10% fetal calf serum, 100 μg/ml gentamicin, and 50 U/ml interleukin 2 (IL-2; Sigma, USA).

Human macrophages (M**ϕ**) were obtained by allowing the monocytes present in the PBMC suspension (1–2 × 10^6^ cells/ml) to adhere as a result of PBMC incubation at 37°C for 2 h; the adherent cells were further differentiated for 7–8 days in the presence of 0.2 μg/ml GM-CSF (Invitrogen, USA), and then added to the growth medium of the composition described above.

Dendritic cells (DCs) were obtained by culturing human monocytes in the presence of 20 ng/ml interleukin 4 (IL-4; Sigma, USA) for 7 days; the monocyte fraction was isolated from PBMC by the MACS (magnetic cell separation) technology, using human CD14 MicroBeads (Miltenyi Biotec, Germany). The antigen-presenting function of DCs was assessed according to the latter’s ability to stimulate allogeneic proliferation of T cells.

The TZM-bl cell line (NIH AIDS Reagent Program No. 8129, USA) was obtained by genetic engineering of HeLa cells; it expresses CD4, CXCR4, and CCR5 and contains the Tat-dependent luciferase reporter gene under the regulatory control of HIV-1 LTR.

293T/17 human kidney epithelial cells were derived from the 293T cell line (ATCC® CRL-11268).

*Env-pseudotyped virus production. *Pseudoviruses were obtained according to the previously described method [34]. 293T/17 cells were seeded at a concentration of 2 × 10^6^ per T-75 flask in a 20-ml DMEM growth medium. After 24 hrs, the cells were transfected with 4 μg of a HIV-1 *env *expression plasmid and 8 μg of an env-deficient HIV-1 backbone vector, pSG3ΔEnv, using the Fugene 6 transfection reagent (Promega, USA). After 4 h of incubation, the transfection medium was replaced with a fresh growth medium. Pseudovirus-containing culture supernatants were collected after 48 h and stored at -80°C. All plasmids were obtained from the international repository of the NIH AIDS Reagent Program.

*TZM-bl assay. *The neutralization analysis was performed using TZM-bl cells according to the Montefiori method [35], which is a modified version of the analysis by Wei et al. [36]. Fresh trypsinized cells were seeded in 96-well plates at a concentration of 1 × 10^4^ cells/well in 100 μl of a DMEM growth medium supplemented with 5 μg/ml DEAE-dextran. Various dilutions of the test compound were added to the cells and incubated for 45–90 min at 37°C. The cells were inoculated with 150,000 RLU (relative luminescent units) of the corresponding pseudovirus. After 48 h of incubation, the cells were washed with the medium and lysed, after which the amount of luciferase was determined in comparison with the virus control using a Victor X3 luminometer (Perkin Elmer). The 50% inhibitory concentration values were calculated using GraphPad Prism 6 by the log function (inhibitor), compared to the normalized response.

*Viruses. *The following HIV-1 strains were used to infect the cells:

HIV-1_BRU_, reference strain of the X4 phenotype, actively replicating in T lymphoblastoid cells and sensitive to azidothymidine (AZT);

HIV-1_AR216_, a clinical isolate of the X4 phenotype (obtained from an HIV-infected patient) adapted to T cell lines and highly resistant to AZT;

HIV-1_Ba-L_, an M-tropic strain of the R5 phenotype;

HIV-1_SF162_, an M-tropic strain of the R5 phenotype;

HIV-1_QH0_, an M-tropic strain of the R5 phenotype (isolated from an HIV-infected patient in Trinidad and Tobago).

To assess the biological activity, the median tissue culture infection dose (TCID_50_) was determined for each viral stock by titration.

**Assessment of cell viability**

Cell viability was determined by the MTT assay based on the ability of live cells to convert the readily soluble yellow 3-(4,5-dimethylthiazol-2-yl)-2,5-diphenyltetrazolium bromide (MTT) into insoluble purple intracellular crystals of MTT-formazan. The conversion efficiency is indicative of the general level of dehydrogenase activity of the cells under study, which is to a certain extent directly proportional to the concentration of viable cells [37]. According to the generally accepted criteria, substances with CC50 ranging from 1 to 10 μg/ml are considered highly toxic; 11 to 20 μg/ml, toxic; 21 to 50 μg/ml, moderately toxic; and 51 to 100 μg/ml, slightly toxic. The substances with CC_50_ > 100 μg/ml are classified as non-toxic.

**Studies of anti-HIV activity**

To determine the antiviral activity, the cells were incubated at variable concentrations of SBF-HS (10, 1, 0.1, and 0.01 μg/ml) at 37°C in 96-well microtiter plates (Corning, USA) for 2 h. The virus was then inoculated at a multiplicity of infection of 100 TCID50. Following the 24-h incubation, the unbound virus was removed by low-speed centrifugation and the cell pellet was re-suspended in a fresh portion of the growth medium. The microplates were monitored for 5 days; the cytopathic effect was assessed according to cell lysis and syncytium formation. The antiviral effect was evaluated according to the decrease in the production of HIV-1 core antigen, p24, which was measured by ELISA. The viral core antigen p24 is a highly conserved major protein universally adopted as a marker of HIV infection. The p24 level was measured using certified commercial test systems manufactured by Bio-Rad (USA) and Vector-Best (Russia).

**Statistical analysis**

Experimental data were obtained in three independent experiments, and the results were shown as the mean ± standard error of the mean (SEM). The significance of the differences between the samples was estimated using the Kolmagorov–Smirnov test and the one-way ANOVA test with Bonferroni adjustment (Origin Pro 2016G, OriginLab Corporation) for experiments with more than two subgroups. The half-maximal inhibitory concentration (IC_50_) and 50% cytotoxic concentration (CC50) were calculated based on the dose-effect curves using the Origin Pro 2016G and Sigma Plot 12.5 software. Differences were considered significant if the calculated *p *value was < 0.05.

## RESULTS AND DISCUSSION

**Characterization of the chemical composition of SBF-HS**

Studies of the elemental composition of SBF-HS were performed in two repetitions. It was determined that SBF-HS is a polyelemental substance with a high content of P and Na (6,000 and 10,029 μg/ml, respectively). It is important to note that the test substance contained almost no heavy metals.

GC-MS analysis showed that fatty and resin acids were the most representative groups in the SBF-HS. Palmitic, oleic, and behenic acids were the predominant fatty acids (1.2 μg/g each). The contents of stearic, arachidic, and lignoceric acids in SBF-HS were lower (0.4 μg/g each). Among the resin acids, three components corresponding to the structural formula of C_20_H_30_O_2_ (1.6 μg/g) were found in the analyzed sample. One of these components can be identified as abietic acid (0.6 μg/g). The other components were isomeric structures having this gross formula. The other two resin acids had the gross formula of . The most characteristic peak in the mass chromatogram corresponded to levopimaric acid (18 μg/g).

We used NMR spectroscopy to compare data on the moiety composition of SBF-HS with the composition of humic acids in coal (*Table 1*). The content of carbonyl, carboxyl, and ester fragments in SBF-HS was similar to that of humic acids in coal. The content of phenolic groups in SBF-HS was slightly higher (11% versus 7–9%). The high C_COO-H_/C_COO-R_ and C_Ar-OH_/C_Ar-OR_ ratios were typical and represented the degree of hydrolysis of the structure. The high content of aromatic fragments was a typical feature of SBF-HS, while the aromatic portion of the structure was characterized by a large number of unsubstituted and O-substituted moieties.

**Table 1:** Moiety composition of SBF-HS

Moiety	Content, %C
C_C=O_	3
C_COO-H_	10
C_COO-R_	2
C_Ar-OH_	11
C_Ar-OR_	7
C_Ar-R_	21
C_Ar-H_	27
C_O-Alk-O_	0
C_-CH-OH_	3
C_-CH2-OH_	6
C_CH3O_	15

**Cytotoxicity of SBF-HS**

In order to assess the cytotoxic effects, the cells were cultured in the presence of varied concentrations of SBF-HS for 3–4 days; the viability was then measured using the MTT assay.

Cytotoxicity studies in three independent experiments demonstrated that SBF-HS was non-toxic to both the CEM-SS cell line and the primary cells (PBMC) (*Table 2*). IC_50_ is the 50% inhibitory concentration (causing 50% suppression of the infection); CC50 is the 50% cytotoxic concentration (causing loss of viability in 50% of cells); SI is the selectivity index calculated as the ratio between CC50 and IC_50_. The values are the means of three measurements, each performed in an independent experiment (antiviral activity was assessed together with toxicity).

**Table 2:** Antiviral activity, cytotoxicity, and selectivity index (SI) of SBF-HS in CEM-SS and primary CD4-positive T cells

Cell	Viral strain	Inhibition of HIV replication, μg/ml			Cytotoxicity CC_50_, μg/ml	SI
		IC_90_	IC_50_ ± SEM	R2		
CEMSS	HIV-1BRU HIV-1AR216	5.4 26.0	0.8 ± 0.3 4.6 ± 0.55	0.87 0.79	708	865 154
PBMC	HIV-1BRU HIV-1AR216	5.3 11.5	0.9 ± 0.2 1.04 ± 0.38	0.91 0.85	631	701 607

**Antiviral activity of SBF-HS in continuous and primary CD4-positive T cells infected with AZT-sensitive or AZT-resistant HIV-1 strains**

The data on the inhibition of the experimental HIV infection indicate that SBF-HS is able to efficiently suppress the infection induced by AZT-sensitive (HIV-1_BRU_) and AZT-resistant (HIV-1_AR216_) virus strains in both continuous and primary cells (*Table 2*). Of note, the concentrations required to achieve 50% suppression of the infection induced by the sensitive strain were almost identical in the continuous and primary cells ( < 1 μg/ml).

The ability to suppress the infection induced by the AZT-resistant strain was cell-dependent: judging from the 50% inhibitory concentration (IC_50_) values, SBF-HS was more efficient in PBMC than CEM-SS cells. The selectivity index (SI) characterizing the clinical promise of the preparation under study was comparably high in PBMC (600–800). This cellular model of an HIV infection is preferable to that based on the use of continuous cell lines. The main reason for the limited use of PBMC in experimental studies of the HIV infection is the lack of standardization and, as a consequence, the chance to encounter a donor with individual resistance to the HIV infection. In order to avoid the latter, we used a mixture of PBMCs isolated from three HIV-seronegative donors.

**Antiviral activity of SBF-HS in PBMC, macrophages (Mϕ), and dendritic cells (DCs)**

We further studied the antiviral activity of SBF-HS in PBMC, macrophages (M**ϕ**), and dendritic cells (DC) infected with the M-tropic HIV-1 strains HIV-1_Ba-L_, HIV-1_SF162_, and HIV-1_QH0_ (*Table 3*).

**Table 3:** Inhibition of viral replication in TZM-bl, PBMC, Mϕ, and DCs infected with HIV-1SF162, HIV-1_QH0_, and HIV-1_Ba-L_

Cells	Virus	Inhibitory concentration, μg/ml			
		IC_90_	IC_80_	IC_50_	R^2*^
PBMC	HIV-1_SF162_ HIV-1_QH0_	5.8 60.0	3.0 20.0	0.9 ± 0.25 9.8 ± 2.9	0.89 0.72
Mϕ	HIV-1_Ba-L_	1.4	0.9	0.3 5± 0.1	0.78
DC	HIV-1_Ba-L_	21.0	12.0	6.8 ± 1.3	0.76
TZM-bl	SF162 pseudovirus QH0 pseudovirus	24.0 60.0	- -	5.0 ± 0.7 5.0 ± 1.2	0.85 0.87

^*^R^2^ values were calculated for IC_50_.

Our studies made it possible to (a) differentially assess the efficacy of SBF-HS in distinct populations of immune cells and (b) generate data demonstrating that the activity of the preparation varies with the cell type.

The infection induced in Mϕ by the R5 strain HIV- 1_Ba-L_ exhibited maximum sensitivity to SBF-HS: 90% and 50% inhibition was observed in the presence of relatively low amounts of the preparation (*Table 3*), whereas 15- to 19-fold higher concentrations were required to achieve the same effect in DCs infected with the same strain. In PBMCs infected with the R5 strain HIV-1_SF162_, 90% inhibition of the infection could be achieved at 6.0 μg/ml SBF-HS. As shown in *Table 1*, almost the same concentration of SBF-HS was sufficient to suppress an HIV infection induced by X4 strains. This observation emphasizes the universality of SBF-HS as an anti-HIV agent, against viruses of both phenotypes (R5 and X4). However, our studies demonstrated that there are HIV-1 strains that exhibit greater resistance to SBF-HS. For example, 10-fold higher concentrations are required to suppress the infection induced in PBMCs by HIV-1_QH0_. The interstrain differences in the genome and pre-existing mutations resulting from the natural polymorphism of HIV-1 may be the likely reasons for the observed discrepancies in SBF-HS activity.

In spite of the differences in IC90 and IC_50_ observed between the studied virus–cell systems, 90% suppression of the infection by SBF-HS was achieved in all cases, which indicates that the preparation protected a considerable percentage of target cells attacked by HIV. Our observation that diverse populations of immune cells differed in the extent of HIV suppression regardless of the tropism and phenotype of the virus suggests that preparation bioavailability may be cell-type-dependent. The differences in infection inhibition in various subpopulations of immune cells, which are independent of the viral phenotype, probably indicate that there also are differences in the bioavailability of the preparation. The unequal penetration of drugs into various cells (tissues) is a fact well-known in the literature. This may result in an insufficient (suboptimal) concentration of the drug to inhibit the virus. Under incomplete suppression of virus replication, favorable conditions for the selective selection of resistant forms are established. This should be taken into account when performing preclinical studies of new drugs. Furthermore, more detailed studies into virus replication inhibitors using individual subpopulations of the immune cells involved in the pathogenesis of HIV are required.

Progressive diminution of subpopulations of immune cells, the hallmark of HIV infection, inevitably results in the deterioration of certain functions of the immune system, of which antigen presentation, stimulation of T cell proliferation, and regulation of antibody production by B cells are the most prominent. This is paralleled by a decrease in the turnover of immune cells. Although the mechanisms underlying the death of immune cells during an acute and chronic HIV infection have not been completely clarified, the data in the literature suggest that apoptosis, cytotoxic T lymphocytes, and the direct cytopathic effects of the virus are the likely factors involved. Our study demonstrates that SBF-HS makes it possible to achieve near-complete suppression of an HIV infection (by more than 90%); thus, a considerable portion of cells in diverse subpopulations will be rescued from depletion, thereby preserving the functionality of the immune system. Yet another important result of our study was the observation that SBF-HS exhibited maximum efficacy in suppressing an HIV infection in Mϕ. Mϕ, as well as memory T cells, serves as a reservoir of the virus, and this may be the main reason why complete eradication of HIV is impossible (and why lifelong administration of anti-HIV therapies is necessary). Some researchers believe that those cells may provide conditions favoring the selection of resistant strains.

Recent data indicate that Th17 cells producing interleukin 17 (IL-17) exhibit maximum susceptibility to the HIV infection and are, therefore, prone to rapid depletion [38]. IL-17 plays a key role in maintaining the intestinal mucosa impermeable [39]. Loss of IL-17 destroys the mucosal barrier and stimulates microbial translocation, which in turn causes HIV-associated immune hyperactivation [40-43]. Our recent understanding of the involvement of phenotypically distinct subpopulations of immune cells in HIV pathogenesis opens up opportunities hitherto unknown for assessing the anti-HIV potential of new compounds.

**Studying the reproducibility of HIV-infection inhibition by SBF-HS**

**Table 4:** Comparative efficiency assessment of six batches of SBF-HS in an experimental HIV infection CEM SS/HIV- 1-Bru

SBF-HS batch No.	Cytotoxicity (CC50 ± SEM), μg/ml	Inhibitory concentration, μg/ml		SI
		IC^90^	IC^50^±SEM	
2660716	1100.0 ± 123	0.90	0.31 ± .012	3548
2640516	1251.0 ± 380	1.10	0.36 ± .001	3475
2690816	985.0 ± 210	0.95	0.34 ± .021	2897
2680716	1230.0 ± 138	1.00	0.38 ± .01	3242
2630416	1150.0 ± 226	0.95	0.35 ± .023	3285
2610316	1159.0 ± 195	0.94	0.31 ± 0.15	3738

**Fig. 1:**
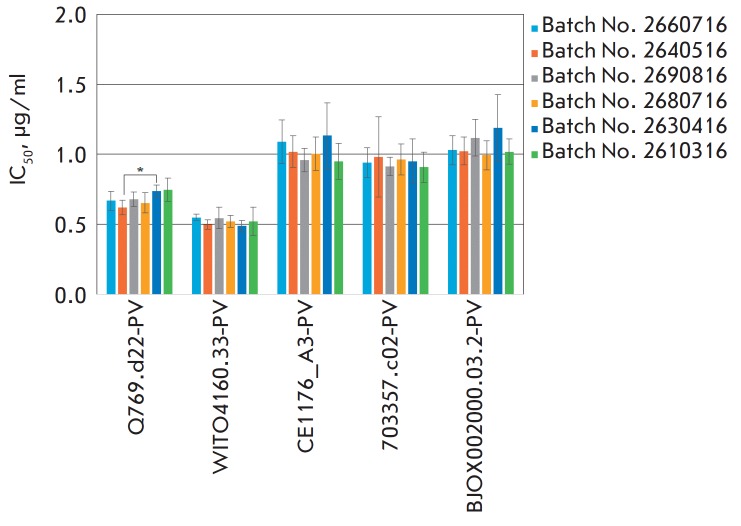
Assessment of the efficacy of SBF-HS under conditions of single-cycle infection (TZM-bl cells and five HIV pseudoviruses). No statistical differences in the inhibition of pseudoviruses were observed between the tested batches of SBF-HS, except for the inhibition of the pseudovirus Q769.d22-PV between batches Nos. 2640516 and 2630416 (*) with p < 0.05

Since stability is an important factor contributing to drug efficacy, we further studied whether the activity of SBF-HS differs between different batches of the preparation. Six batches were examined, and the activity was virtually the same in each case: the HIV infection was inhibited in a dose-dependent manner over the same range of concentrations and with a high SI in diverse virus–cell models HIV-1/Bru and TZM-bl–HIV pseudoviruses (*Table 4, Table 5* and *Fig. 1*).

**Table 5:** Panel of the HIV-1 pseudoviruses used for infecting TZM-bl cells (single-cycle infection)

Virus	Origin (country)	Subtype	Infection stage	Transmission
Q769.d22-PV	Kenya	A	Acute/early	Sexual
WITO4160.33-PV	USA	B	II	Sexual
CE1176_A3-PV	Malawi	C	I/II	transmitted founder virus Sexual
703357.c02-PV	Thailand	CRF01_AE	I/II	Sexual
BJOX002000.03.2-PV	China/Beijing	CRF07_BC	I/II	IDU

Pronounced suppression of single-cycle infection by SBF-HS was observed in three independent experiments where TZM-bl cells were infected with pseudoviruses differing in the origin of *env *sequences (which belonged to the A, B or C HIV subtype and two circulating HIV recombinants). Expression vectors for pseudovirus production were selected from the standard panel of HIV-1 reference strains (NIH AIDS Research and Reference Reagent Program; NIH ARRRP). As demonstrated in *Fig. 1*, the IC_50_ values for the pseudoviruses Q769.d22, WITO4160.33, CE1176_A3, 703357.c02, and BJOX002000.03.2 fall within the ranges 0.62–0.75, 0.49–0.55, 0.95–1.13, 0.91–0.98, and 0.99–1.19 μg/ml, respectively.

## CONCLUSION

In conclusion, it is obvious that SBF-HS was capable of efficiently suppressing an experimental HIV-infection and protecting cells from several distinct HIV subtypes and circulating recombinant forms. Assessment of the biological properties of six distinct batches of the preparation demonstrated that the fractionation technique used to obtain standardized preparations of SBF-HS was reliable.

Humic substances formed via the decay of dead organisms constitute one of the vast reservoirs of organic carbon. In 1988, during the International Humic Substances Society meeting, it was suggested that humic substances might offer significant promise as drugs for treating diverse diseases [44]. As of today, there is ample evidence of the rather unique properties of humic substances, which exhibit anti-inflammatory, wound-healing, antifungal, bactericidal, and even antineoplastic activities. In spite of those findings, it is not as if humic substances have become the subject of major scientific investigations. Only two companies (one in the United States and one in Russia) have so far succeeded in developing humic substance-based drugs and in completing all the requisite preclinical and clinical trials of their safety and efficacy [30]. This work supplements our knowledge base pertaining to the possible therapeutic utilities of humic substances, which are thereby extended to the treatment of an HIV infection.

## Abbreviations

HIV: human immunodeficiency virus
SBF-HS: solubilized butanol fraction of humic substances
HPLC: high-performance liquid chromatography
NMR: nuclear magnetic resonance
Mϕ: macrophages
DC: dendritic cells
PBMC: peripheral blood mononuclear cells
AZT: azidothymidine
CTL: cytotoxic lymphocytes
